# Cerebral air embolism: neurologic manifestations, prognosis, and outcome

**DOI:** 10.3389/fneur.2024.1417006

**Published:** 2024-06-19

**Authors:** Vladimír Červeňák, Vít Všianský, Martina Cviková, Jaroslav Brichta, Jan Vinklárek, Jakub Štefela, Michal Haršány, Michal Hájek, Roman Herzig, Dávid Kouřil, Veronika Bárková, Pavel Filip, Petr Aulický, Viktor Weiss

**Affiliations:** ^1^Department of Radiology, Faculty of Medicine, St. Anne's University Hospital, Masaryk University, Brno, Czechia; ^2^Department of Neurology, Faculty of Medicine, St. Anne's University Hospital, Masaryk University, Brno, Czechia; ^3^Center for Hyperbaric Medicine of Faculty of Medicine University of Ostrava and Ostrava City Hospital, Ostrava, Czechia; ^4^Department of Neurology, Faculty of Medicine, Charles University, Hradec Králové, Czechia; ^5^Department of Neurology, Comprehensive Stroke Center, University Hospital Hradec Králové, Hradec Králové, Czechia; ^6^Research Institute for Biomedical Science, Hradec Králové, Czechia; ^7^Department of Neurology, Blansko Hospital, Blansko, Czechia; ^8^Hospital Pharmacy, Department of Clinical Pharmacy, St. Anne's University Hospital, Brno, Czechia; ^9^Department of Neurology, First Faculty of Medicine and General University Hospital, Charles University, Prague, Czechia; ^10^Center for Magnetic Resonance Research (CMRR), University of Minnesota, Minneapolis, MN, United States; ^11^Hospital of the Brothers of Charity Brno, Brno, Czechia

**Keywords:** air embolism, cerebral embolism, cerebral stroke, hyperbaric oxygen therapy, neurological emergency

## Abstract

**Background:**

Cerebral air embolism (CAE) is an uncommon medical emergency with a potentially fatal course. We have retrospectively analyzed a set of patients treated with CAE at our comprehensive stroke center and a hyperbaric medicine center. An overview of the pathophysiology, causes, diagnosis, and treatment of CAE is provided.

**Results:**

We retrospectively identified 11 patients with cerebral venous and arterial air emboli that highlight the diversity in etiologies, manifestations, and disease courses encountered clinically. Acute-onset stroke syndrome and a progressive impairment of consciousness were the two most common presentations in four patients each (36%). Two patients (18%) suffered from an acute-onset coma, and one (9%) was asymptomatic. Four patients (36%) were treated with hyperbaric oxygen therapy (HBTO), high-flow oxygen therapy without HBOT was started in two patients (18%), two patients (18%) were in critical care at the time of diagnosis and three (27%) received no additional treatment. CAE was fatal in five cases (46%), caused severe disability in two (18%), mild disability in three (27%), and a single patient had no lasting deficit (9%).

**Conclusion:**

Cerebral air embolism is a dangerous condition that necessitates high clinical vigilance. Due to its diverse presentation, the diagnosis can be missed or delayed in critically ill patients and result in long-lasting or fatal neurological complications. Preventative measures and a proper diagnostic and treatment approach reduce CAE’s incidence and impact.

## 1 Introduction

Cerebral air embolism (CAE) occurs when gas bubbles enter the arterial or venous system of the brain, potentially obstructing blood flow and resulting in brain injury. It is an uncommon finding, usually iatrogenic, occurring with an unknown incidence and prevalence. There are two main types of CAE, cerebral venous air embolism (CVAE), and cerebral arterial air embolism (CAAE; sometimes also abbreviated as CAGE—cerebral arterial gas embolism). CVAE occurs when gas bubbles enter the venous system of the brain, most often retrogradely through the jugular veins. The bubbles can block veins leading to brain edema, venous infarction, and intracranial hypertension. CAAE involves air entry into the arterial supply of the brain, usually through catheterization or surgical procedures. When air bubbles occlude arteries supplying the brain tissue, it can lead to infarction and neurological deficit.

In this text, we will summarize the current understanding of the pathophysiology, risk factors, diagnosis, and management of these two types of CAE and present 11 cases from our clinical experience that highlight the diversity of etiologies, clinical presentations, imaging findings, and prognosis of these potentially deadly events.

## 2 Materials and methods

We performed a retrospective chart review of patient records from a comprehensive stroke center and from an HBOT center situated in Central Europe (St. Anne’s University Hospital and Center for Hyperbaric Medicine of Faculty of Medicine University of Ostrava and Ostrava City Hospital) from January 1, 2010, to November 1, 2023.

## 3 Results

**Figure 1:**
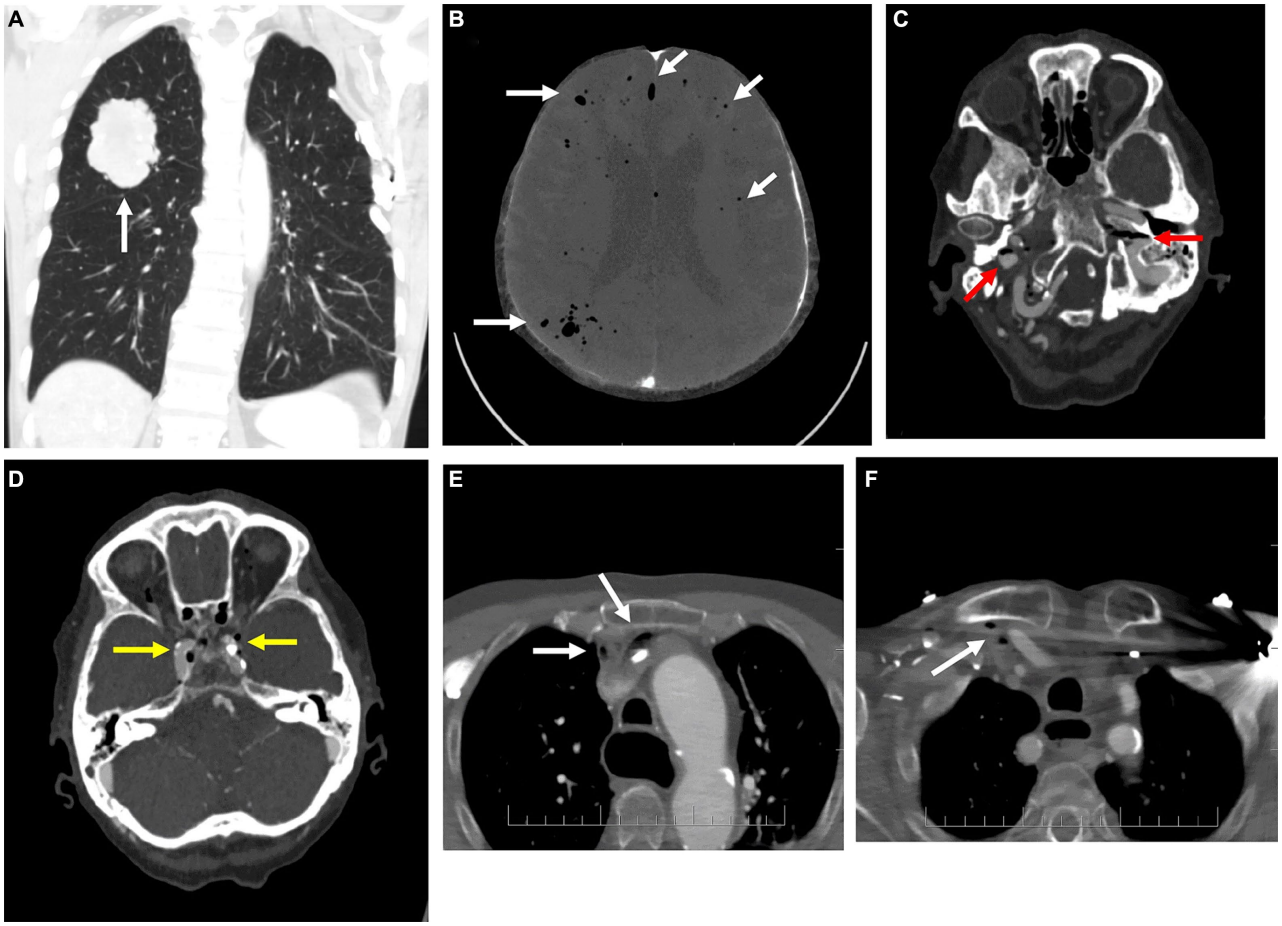
Imaging for cases 1 **(A,B)** and 2 **(C–F)**. **(A)** Coronal plane of the native CT of the lungs showing a typical image of a primary lung tumor in the upper right lobe. **(B)** Axial CT scan of the brain in Minimum intensity projection (MinIP) shows the number of air bubbles in the cerebral circulation (white arrows, MinIP is an imaging technique used to identify low-density structures within a specific volume). **(C,D)** Axial contrast-enhanced CT scans of the brain with gas bubbles in the internal jugular veins (red arrows) and cavernous sinuses bilaterally (yellow arrows). **(E,F)** Axial contrast-enhanced CT scans of the upper thorax with air bubbles in the right subclavian vein and right and left brachiocephalic veins (white arrows).

**Figure 2:**
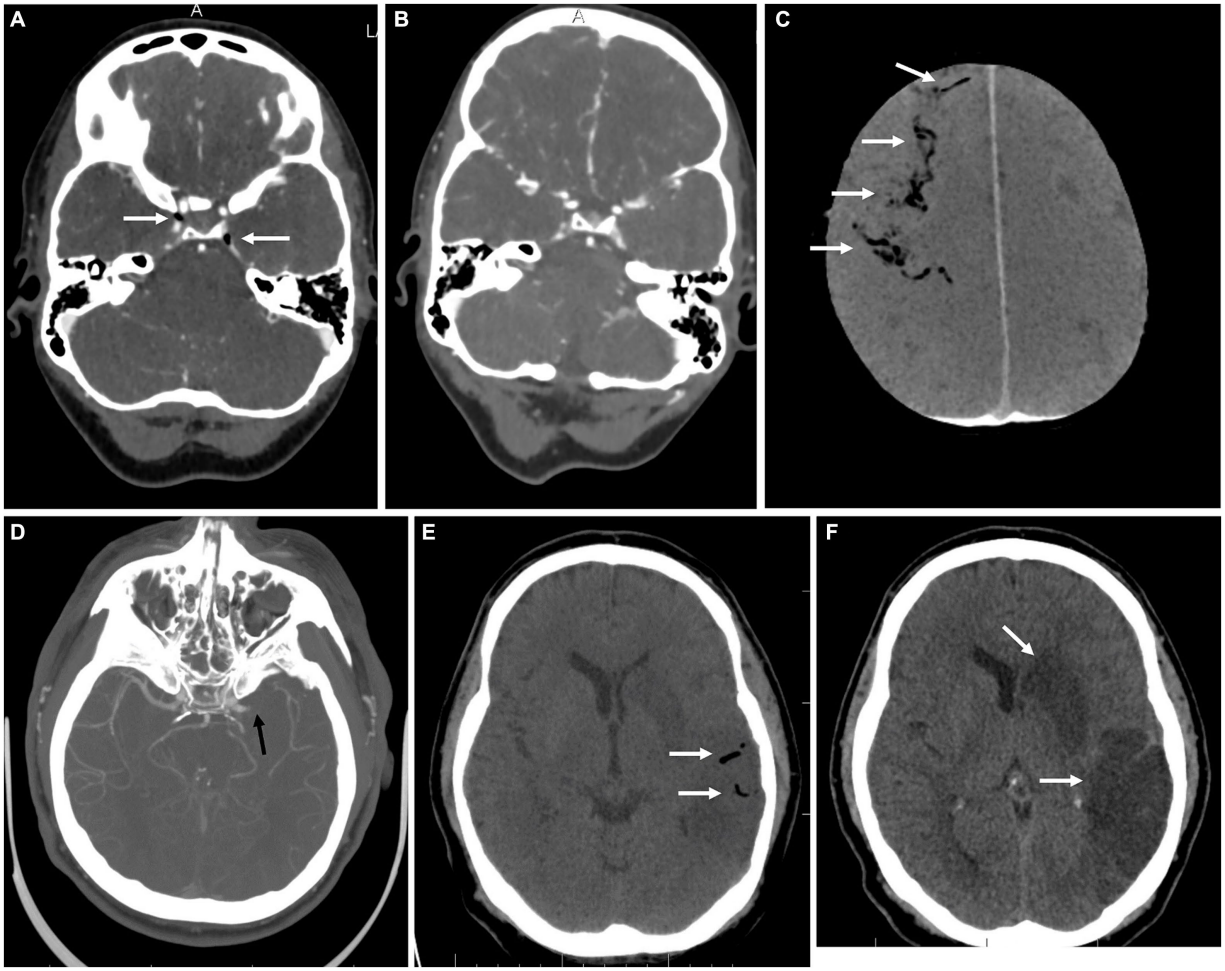
Imaging for case 3. **(A)** Axial native CT scan of the lungs showing a solid nodulus with an inserted percutaneous biopsy needle (white arrow). **(B)** Follow-up native CT scan of the lungs after the biopsy with a small pneumothorax and intraparenchymal hemorrhage behind the lesion due to damage to the integrity of the vascularity (white arrow). **(C)** Axial native CT scan of the brain in MinIP shows a smaller amount of air bubbles in the cerebral circulation (white arrows). **(D)** Axial contrast-enhanced CT scan of the distal part of the neck with air in the left internal jugular vein (white arrow). **(E)** Follow-up axial CT scan of the brain in MinIP shows a single residual air bubble in the region of the falx cerebri (white arrow). **(F)** Axial DWI brain scan showing no diffusion restriction and no evidence of acute ischemia.

**Figure 3:**
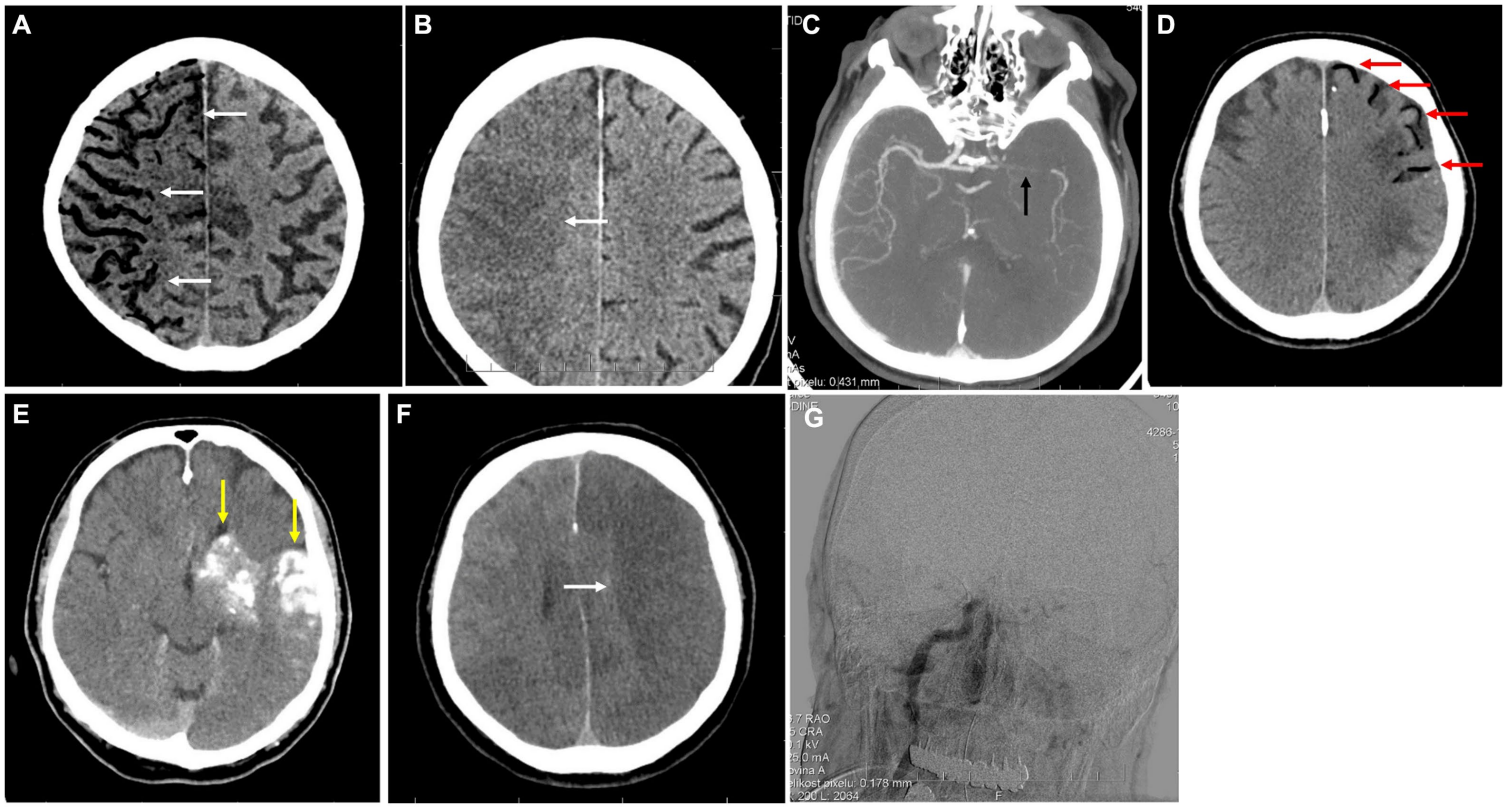
Imaging for cases 4 **(A,B)** and 5 **(C–G)**. **(A)** Axial native CT scan of the brain in MinIP shows a very significant amount of air bubbles in the cerebral circulation in the right hemisphere (white arrows). **(B)** Axial CT scan of the brain reveals extensive delineated ischemia of almost the entire right cerebral hemisphere (white arrow). **(C)** Axial CTA of the brain showing occlusion of the M1 segment of the left middle cerebral artery (black arrow). **(D)** Axial native CT scan with a significant volume of air in the brain circulation on the left side (red arrows). **(E)** Native axial CT scan with contrast agent extravasation and intracerebral and subarachnoid hemorrhage (yellow arrows). **(F)** Axial native CT scan of the brain with developing extensive acute ischemia in the left middle cerebral artery territory (white arrow). **(G)** Cerebral panangiography without intracranial arterial flow, demonstrating brain death.

**Figure 4:**
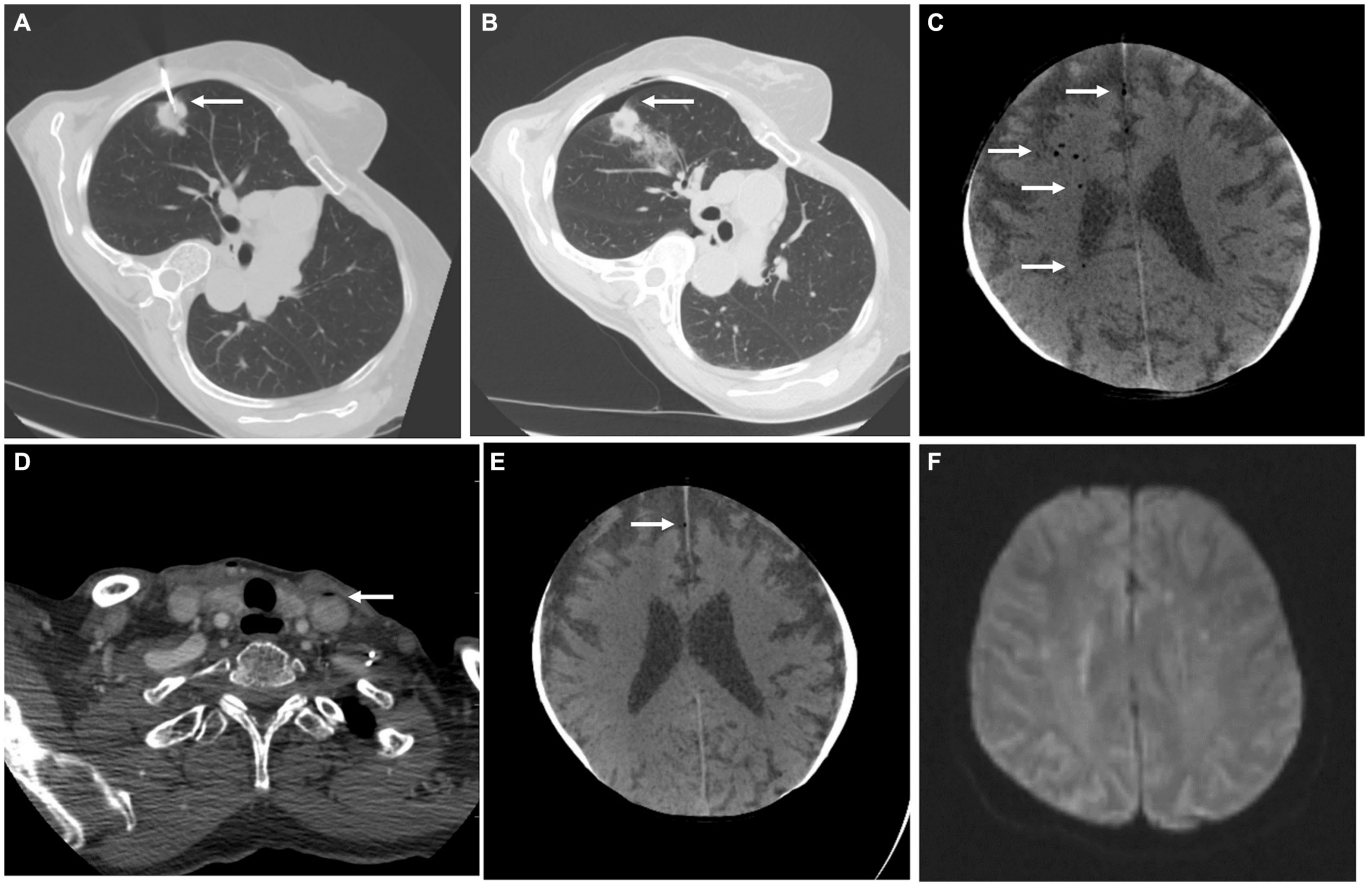
Imaging for cases 6 **(A,B)**, 7 **(C)**, and 11 **(D–F)**. **(A)** Axial CTA scan of the brain with air bubbles in cavernous sinuses (white arrows). **(B)** CTA of the brain vasculature on the next day showing complete regression of the air emboli. **(C)** Axial CT scan of the brain in MinIP shows a very significant amount of air bubbles in the cerebral circulation of the right hemisphere (white arrows). **(D)** Axial brain CTA with occlusion of the M1 segment of the left middle cerebral artery (black arrow). **(E)** Axial native CT scan of the brain in MinIP shows a significant amount of air bubbles in the cerebral circulation in the left hemisphere (white arrows). **(F)** Native axial CT scan of the brain with developing extensive ischemia in the left middle cerebral artery territory, temporal, and occipital region (lower white arrow), and basal ganglia (upper white arrow). Air bubbles have disappeared.

A total of 11 patients with CAE were identified. A summary of the included cases is presented in Table 1 and Figures 1–4.

**Table 1:** Summary of presented cases.

Patient	Medical history	Presentation	Imaging findings	Cause	Treatment	Outcome
1: 69-year-old man	Ischemic heart disease, atrial fibrillation, peripheral artery disease, and smoking.	Progressive impairment of consciousness during ICU stay 8 days after a lung tumor resection.	Pulmonary lesion before resection (Figure 1A). Air in cerebral circulation identified 8 days after the surgery (Figure 1B).	Lung tumor resection (air bubbles originating from the compromised vasculature at the site of the lung tumor resection 6 days after the surgery) or a possible central venous catheter disconnection in the postsurgical period (since the event occurred with a significant time delay from the resection).	Critical care (mechanical ventilation, multiple organ support therapy).	Death
2: 86-year-old woman	Ischemic strokes in both internal carotid artery territories, atrial fibrillation, ischemic heart disease, and type 2 diabetes mellitus.	Stroke (acute worsening of preexisting left-sided motor deficit).	Air in bilateral jugular veins and cavernous sinuses (Figures 1C,D) as well as in the right subclavian vein and right and left brachiocephalic veins (Figures 1E,F).	Contrast agent injection admixed with air during CT angiography (CTA).	None, due to the critical condition of the severely disabled patient.	Severe disability, end-of-life care
3: 81-year-old woman	None	Transient loss of consciousness and non-dominant middle cerebral artery stroke (left-sided hemiplegia, central lesion of the left facial nerve and left-sided homonymous hemianopsia, and eye deviation to the right side and neglect syndrome) immediately after a percutaneous lung biopsy.	Pneumothorax and intraparenchymal lung hemorrhage after lung biopsy (Figures 2A,B). Afterward, the air bubbles in the right middle cerebral artery and left carotid artery were identified (Figures 2C,D). Follow-up head CT on the next day showed regression of the intraarterial air bubbles (Figures 2E,F).	Percutaneous lung biopsy	High-flow oxygen therapy without HBOT. Targeted rehabilitation for the neurological deficit.	Mild disability (mild left-sided hemiparesis)
4: 81-year-old man	Type 2 diabetes mellitus, peripheral artery disease	Progressive impairment of consciousness into a coma during ICU stay for respiratory insufficiency.	Air emboli throughout the right cerebral vasculature with acute ischemic changes (Figure 3A). Follow-up CT scan after 3 days and HBOT therapy showed extensive ischemia in the right cerebral hemisphere, the air has been absorbed (Figure 3B).	Central venous catheter disconnection while receiving nursing care.	Positioning, high-flow oxygen therapy, HBOT, mechanical ventilation	Death
5: 68-year-old man	Arterial hypertension, hyperlipidemia	First admitted for a sudden onset of aphasia, which resolved during transport to the hospital. A few hours later, the patient suddenly developed global aphasia, eye deviation to the left, and severe right-sided hemiparesis. After mechanical thrombectomy for left-sided M1 occlusion, the patient deteriorated into a coma and developed left-sided mydriasis.	A follow-up CT scan after the mechanical thrombectomy showed arterial air embolism in the right hemisphere along with intracerebral hemorrhage and subarachnoid hemorrhage (Figures 3D,E). On the next day, the patient fulfilled the criteria for brain death (Figures 3F,G).	Mechanical thrombectomy	High-flow oxygen therapy, critical care (no HBOT due to critical condition).	Death
6: 24-year-old woman	Migraine without aura	Presented to the ER with a transient paresthesias of her left face spreading to the left upper limb and lasting about 45 min, followed by her usual migraine headache. The CVAE was likely asymptomatic. All symptoms were resolved by the next day.	A small amount of air in bilateral cavernous sinuses (Figure 4A). Follow-up CTA scan on the next day showed a complete regression of the air bubbles (Figure 4B).	Placement/flushing of a peripheral intravenous catheter.	None	No deficit
7: 71-year-old man	Ischemic heart disease	Presented to the ER with acute onset chest pain and dyspnea. Coronarography showed critical stenosis of the left anterior descending artery, which was treated using a drug-eluting stent. During the procedure, due to pulmonary edema, the patient developed respiratory failure and was intubated. During the ICU stay, his consciousness progressively deteriorated.	A head CT scan 7 days after admission showed a significant air embolism in the right cerebral vasculature with diffuse brain edema (Figure 4C)	Unknown (possibly coronarography, embolectomy, and central venous catheter malfunction).	Critical care (mechanical ventilation, multiple organ support therapy)	Death
8: 70-year-old man	Ischemic heart disease, chronic obstructive pulmonary disease, and arterial hypertension.	Somnolence, global aphasia, quadriparesis, head, and eye deviation to the right after the placement of a central venous catheter into the right internal jugular vein.	Venous air embolism in the right frontal region.	Malfunction of a central venous catheter	HBOT	Mild disability (left-sided hemiparesis and slight left-sided gaze palsy)
9: 31-year-old man	Gastric adenocarcinoma (admitted for elective resection)	Epileptic seizure followed by coma and respiratory arrest after central venous catheter removal before planned release.	Diffuse air emboli in the brain vasculature with brain edema.	Central venous catheter extraction	HBOT	Death
10: 24-year-old woman	Pineal gland region cyst (admitted for elective resection)	Progressive impairment of consciousness and quadriparesis several hours after the surgery.	Left cerebellar hemisphere ischemia with air emboli.	Pineal cyst surgery	HBOT	Mild disability (mild left-hand weakness)
11: 53-year-old man	Arterial hypertension	Admitted for sudden-onset aphasia and right-sided hemiplegia with a central lesion of the right facial nerve. After intravenous thrombolysis, the patient underwent mechanical thrombectomy, which achieved complete reperfusion (TICI 3).	Initial CT and CTA scan showed left middle cerebral artery M1 segment occlusion (Figure 4D) with brain ischemia in the corresponding vascular territory (ASPECTS 7). CT imaging 24 h after stroke onset revealed a more extensive infarction in the right temporal lobe along with a significant amount of air in the left hemisphere cerebral vessels (Figures 4E,F).	Mechanical thrombectomy	None (due to the extensive ischemia, additional treatment for the CAE would not confer clinical benefit)	Severe disability (aphasia and right-sided hemiplegia)

Concise descriptions of clinical cases of CAE with their medical history, presentation, imaging findings, cause, treatment, and outcomes.

The median age of the included patients was 69 years (24–86 years). Four (36%) of the patients were women. The onset of symptoms in CAE is usually acute. Six patients in our cohort (55%) had an acute presentation, of which four (36%) were stroke syndromes and two (18%) were acutely comatose.

Less commonly, progressive worsening and fluctuation of symptoms are observed. From our observation, patients in intensive care suffered from a progressive deterioration more often than others, three of them (75%) were already in the ICU for unrelated reasons before the first symptoms of CAE and one (25%) was post-surgery. This can make the diagnosis significantly more difficult.

In our patient cohort, death was the most common outcome of CAE at 46% and only a single patient (9%) was without any disability at follow-up. CAE, particularly CVAE, can also be completely asymptomatic, being diagnosed as an incidental finding on brain imaging. This was the case for one of our included patients (9%). Only four patients (36%) were treated with HBOT, two (18%) received high-flow oxygen therapy and positioning without HBOT, two (18%) were in critical care during the CAE diagnosis and three (27%) received no additional therapy for CAE. The most common outcome of a CAE was death, which occurred in five cases (46%). Two patients (18%) had a severe disability at the last follow-up and three (27%) had a minimal disability. A single patient (9%) was completely without neurological deficit.

## 4 Discussion

### 4.1 Pathophysiology

In CAAE, air enters the arterial circulation either directly through a breach in an arterial wall or indirectly through an intracardiac or pulmonary shunt from the venous circulation. When this happens, the air follows the blood flow and occludes one of the major intracranial arteries and/or blocks terminal arterioles as the smaller bubbles cannot continue past the decreasing diameter of the lumen. Ultimately, the result of this process is brain tissue ischemia in the affected vascular territories.

Venous emboli can cause CAAE through paradoxical embolization, most commonly via a patent foramen ovale, which is present in about 24.2% of the general population (1). In critical care patients, barotrauma can occur as a complication of mechanical ventilation and may manifest as pulmonary interstitial emphysema, pneumothorax, and pneumomediastinum (2). Systemic air embolism has been recognized to occur as a complication of mechanical ventilation, during which air has been detected in the cerebral arterial circulations (3, 4).

The pathophysiology of CVAE is more complicated. Air can enter intracranial veins or venous sinuses when they are directly compromised, for example during a neurosurgical procedure. Sometimes, no clear cause of the CVAE is identified (5). The most common etiology of CVAE, however, is a central venous catheter placement, removal, or malfunction (6). As the blood in the venous system flows away from the brain toward the heart, one could expect the air bubbles to follow this flow and end up lodged in the pulmonary circulation, which does happen to some extent in every case of venous air embolism. However, it has been experimentally demonstrated that air bubbles can, under the right conditions, travel retrogradely toward the intracranial veins due to their low specific weight (7). The main factors enabling this retrograde flow in CVAE are insufficiency of the jugular vein valves, hypovolemia leading to low venous pressure, upright position (>45°) causing a lower pressure gradient for the gas bubbles, and, increased intrathoracic pressure, such as during mechanical ventilation.

Depending on the amount of air that enters the cerebral venous system, CVAE can cause various degrees of blood stasis and ultimately venous brain infarction. Additionally, air bubbles trigger an inflammatory reaction of the endothelium that can lead to activation of the coagulation system further exacerbating the venous congestion (8). In some cases, when the amount of air is not significant, CVAE can be asymptomatic.

The rate of air embolism resorption depends on air volume, bubble shape (with an elongated linear bubble taking longer to resorb compared to a spherical one), and blood flow velocity. CAE should resorb completely in minutes to hours given that no more air is entering the circulation (9).

Specific causes of CAAE and CVAE are summarized in Table 2. Most common etiology of CAE in our patients was central venous catheter related (either disconnection, malfunction or during extraction) which occurred in three cases. Two patients had a CAE following mechanical thrombectomy. In these patients, no clear reason for an air embolism was recognized during the procedure. To decrease the chance of CAE during mechanical thrombectomy, it is important to use an air filter during the contrast injection, which should be done slowly, as well as priming the catheter to eliminate all air in the circuit.

**Table 2:** Summary of CAAE and CVAE etiologies.

CAAE etiologies	CVAE etiologies
Paradoxical embolization from the venous system (10)	Central venous catheter placement, malfunction, or removal (7, 10, 11)
Percutaneous lung needle biopsy (12)	Neurosurgical procedures (13)
Chest, head, and neck trauma (14, 15)	Head and neck trauma
Cardiopulmonary resuscitation (16)	Hydrogen peroxide irrigation during surgery (17–19)
Positive pressure ventilation (20)	Hemodialysis (21–23)
Mechanical thrombectomy (24)	
Cardiac catheterization and cardioverter placement (21)	
Cardiopulmonary bypass (25)	
Arterial line flushing (26)	
Hemodialysis (27)	
Bronchoscopy (28)	
Gastrointestinal endoscopy (29)	
Diving barotrauma (30)	

Described etiologies of CAAE and CVAE with corresponding case reports and case studies.

Other causes, including a lung tumor resection, percutaneous lung biopsy, pineal cyst surgery, peripheral intravenous catheter placement, and contrast agent injection admixed with air, were observed a single time each. All CAEs were iatrogenic.

### 4.2 Clinical manifestations

Cerebral air embolism can cause a broad spectrum of neurological symptoms, which include encephalopathy with a varying degree of mental status alteration and impairment of consciousness (5, 10, 21, 23), large vessel occlusion stroke (29), focal neurological deficits (e.g., aphasia, hemiparesis, facial droop, and hemianopsia) (10), symptomatic epileptic seizures (23) or, headache (31). This rather general list of symptoms means that CAE can mimic many other neurological conditions, such as stroke from other causes, intracranial hemorrhage, epilepsy, etc. The main distinguishing factor is the temporal relationship of the onset of symptoms to the causative event allowing air to enter the circulation. In addition to neurological symptoms, CAE can also cause cardiovascular and respiratory manifestations due to coinciding air embolisms in the pulmonary and/or cardiac circulation.

In some cases, the event can be self-limiting, though a lasting neurological deficit is often present, particularly in larger infarctions and in patients significantly disabled at the time of presentation. A fatal course is also not unusual (11, 21, 32).

### 4.3 Diagnosis

An accurate diagnosis of cerebral air embolism requires a high index of suspicion based on clinical history and presentation. A history of recent trauma, surgery, medical procedures, or risks that expose the patient to air suggests the possibility of cerebral air embolism. All of our included patients had a presumed iatrogenic cause.

A “mill-wheel” murmur can be appreciated on heart auscultation with large intracardiac air emboli (33). Signs of acute respiratory failure, pulmonary edema, and shock can also be apparent during the physical examination. Often, however, the physical examination is unrevealing.

Transthoracic and transesophageal echocardiography (TEE) have been used to document the presence of air in cardiac chambers as well as air in the great veins. They may also show evidence of acute right ventricular dilation and pulmonary artery hypertension (34). TEE and transcranial Doppler ultrasonography are useful adjunct tests that can detect intracardiac shunts and air bubbles in intracranial vessels, respectively.

Imaging studies play a key role in establishing the diagnosis. Computed tomography (CT) is highly sensitive for the detection of gas in the vessels which appears as highly hypodense areas (the radiodensity of air is defined as −1,000 Hounsfield units). However, as the gas can be resorbed rather rapidly, the initial CT may not show evidence of air directly but only the consequences of CAE, such as cerebral infarction or brain edema. Minimum intensity projection (MinIP) is a visualization method that selectively detects the most hypodense structures in a given volume. In CAE, this can be employed to highlight air bubbles in the cerebral circulation (Figure 1B).

Magnetic resonance imaging (MRI) can also be helpful in the diagnosis of CAE, but it is usually reserved for assessment of consequences of CAE or differential diagnosis, not for directly proving the presence of air, as the CT is usually the more readily available and faster to perform the diagnostic method. In our cohort, CT was used in all patients. The most important MRI sequence in CAE diagnosis is diffusion-weighted imaging (DWI), which shows areas of brain infarction with very high sensitivity.

### 4.4 Treatment and management

The primary strategy in CAE management is prevention, which involves strict adherence to procedural guidelines, especially during invasive medical procedures. In case a CAE is suspected, the first step is to prevent further air from entering the circulation, e.g., by putting pressure on the open wound.

A patient with venous air embolization including CVAE should be immediately placed into the left lateral decubitus position (Durant’s maneuver), Trendelenburg position, or left lateral decubitus head-down position (35). Trendelenburg position should avoid additional bubbles from migrating into the brain vasculature by directing air bubbles upwards. In the case of a massive venous air embolism, obstruction of the right ventricle outflow tract by air can lead to shock or cardiac arrest. The left lateral decubitus position is supposed to prevent air from obstructing the right ventricular outflow tract by moving the bubbles into the right atrium (36). However, there are some controversies surrounding these positional maneuvers (especially in the case of the head-down position) due to their potential for exacerbating cerebral edema and increasing intracranial pressure.

For CAAE, the right lateral decubitus position could in theory prevent air bubbles from entering the left ventricular outflow tract by trapping them in the upper portion of the left ventricle (37). Trendelenburg position worsens cerebral edema and intracranial hypertension and therefore should not be used in these cases. The most common recommendation is that a patient with arterial air embolism should be placed in the supine position (35).

High-flow 100% oxygen therapy should be started immediately. The supplemental oxygen increases the partial pressure of oxygen and decreases the partial pressure of nitrogen in the blood. This causes the diffusion of nitrogen from inside the air bubble into the blood, which reduces bubble size and accelerates air resorption. Simultaneously, additional steps to stabilize the patient should be taken as necessary, including airway (tracheal intubation), breathing (mechanical ventilation), and circulation (intravenous fluid resuscitation, vasopressor therapy) management. In comatose patients or those with symptoms of convulsive or non-convulsive status epilepticus, electroencephalography monitoring and treatment with antiseizure medication is indicated.

The definitive treatment for cerebral air embolism is hyperbaric oxygen therapy (HBOT). When available, HBOT should be administered to patients with evidence of hemodynamic or cardiopulmonary compromise, as well as to those with neurologic deficits or other evidence of end-organ damage (38, 39). HBOT should be administered as soon as possible (within the first 4–6 h after symptom onset) as its efficacy diminishes with time (6). HBOT provides oxygen at pressures greater than atmospheric pressure and at 100% concentration so that very high levels of systemic hyperoxia can be achieved. This degree of hyperoxia allows enormous gradients for nitrogen to be displaced from inside the air bubble, which in turn, reduces air bubble size and the degree of arterial blood flow obstruction. Moreover, by increasing the pressure, the volume of the air bubbles is decreased as per Boyle’s law (volume of gas has an inverse relationship with pressure at a constant temperature) and the concentration of dissolved oxygen in plasma is increased which can enhance oxygen supply to ischemic tissues. The benefits of HBOT therapy should be weighed against the risk of death during transfer (40). In our cohort, four patients (36%) in critical state were considered to be too high risk for a transfer to an HBOT facility.

Adjunct treatment with lidocaine infusion is suggested by the European Consensus Conference on Hyperbaric Medicine (41). Lidocaine might confer some neuroprotective effects, although robust data on this treatment are not available (42).

The use of anticoagulation and antiplatelet agents in cerebral air embolism is controversial. In theory, these medications may prevent further thrombosis on the surface of the air bubble. However, there is no robust evidence to suggest a significant improvement in outcomes and the air emboli should resorb rapidly. As such, the decision to use these therapies should be individualized, taking into account the risk of hemorrhage, especially in cases of trauma-related embolism.

## 5 Conclusion

Cerebral air embolism, although rare, is a critical and potentially fatal complication that warrants high clinical vigilance. It presents with diverse symptoms including acute or progressive impairment of consciousness, seizures, or stroke, and can occur in various settings, often following invasive procedures, trauma, or other specific medical conditions. Timely diagnosis is contingent on the index of clinical suspicion and the patient’s medical history. Imaging techniques, particularly CT, play an essential role in confirming the diagnosis. The treatment strategy usually focuses on preventive measures, immediate stabilization of the patient, and hyperbaric oxygen therapy as a definitive treatment.

## Data availability statement

The original contributions presented in the study are included in the article/supplementary material; further inquiries can be directed to the corresponding author.

## Ethics statement

The studies involving humans were approved by the Ethics committee of St. Anne’s University Hospital. The studies were conducted in accordance with the local legislation and institutional requirements. The participants provided their written informed consent to participate in this study. Written informed consent was obtained from the individual(s) for the publication of any potentially identifiable images or data included in this article.

## Author contributions

VČ: Writing – review & editing, Writing – original draft, Visualization, Methodology, Data curation, Conceptualization. VV: Writing – review & editing, Writing – original draft, Visualization, Investigation, Data curation, Conceptualization. MC: Writing – review & editing, Data curation. JB: Writing – review & editing, Data curation. JV: Writing – review & editing, Data curation. JŠ: Writing – review & editing, Data curation. MHar: Data curation, Writing – review & editing. MHáj: Visualization, Writing – review & editing, Data curation. RH: Writing – original draft, Supervision, Funding acquisition, Writing – review & editing, Data curation. DK: Writing – review & editing, Data curation. VB: Writing – review & editing, Data curation. PF: Visualization, Writing – review & editing, Data curation. PA: Supervision, Data curation, Writing – review & editing. VW: Writing – review & editing, Writing – original draft, Visualization, Resources, Project administration, Methodology, Funding acquisition, Data curation.

## References

[1] Koutroulou I Tsivgoulis G Tsalikakis D Karacostas D Grigoriadis N Karapanayiotides T. Epidemiology of patent foramen Ovale in general population and in stroke patients: a narrative review. Front Neurol. (2020) 11:281. doi: 10.3389/fneur.2020.00281, PMID: 32411074 PMC7198765

[2] Haake R Schlichttg R Ulstad DR Henschen RR. Barotrauma. Chest. (1987) 91:608–13. doi: 10.1378/chest.91.4.608 3549176

[3] Ibrahim AE Stanwood PL Freund PR. Pneumothorax and systemic air embolism during positive-pressure ventilation. Anesthesiology. (1999) 90:1479–81. doi: 10.1097/00000542-199905000-00035, PMID: 10319799

[4] Marini JJ. Systemic gas embolism complicating mechanical ventilation in the adult respiratory distress syndrome. Ann Intern Med. (1989) 110:699–703. doi: 10.7326/0003-4819-110-9-699, PMID: 2930107

[5] Costa Carneiro A Diaz P Vieira M Silva I Custodio M Silva M. Cerebral venous air embolism: a rare phenomenon. Eur J Case Rep Intern Med. (2019) 6:1. doi: 10.12890/2019_001011 PMC6372048 30756075

[6] Blanc P Boussuges A Henriette K Sainty J Deleflie M. Iatrogenic cerebral air embolism: importance of an early hyperbaric oxygenation. Intensive Care Med. (2002) 28:559–63. doi: 10.1007/s00134-002-1255-0, PMID: 12029402

[7] Fracasso T Karger B Schmidt PF Reinbold WD Pfeiffer H. Retrograde venous cerebral Air embolism from disconnected central venous catheter: an experimental model: Retrograde venous cerebral air embolism. J Forensic Sci. (2011) 56:S101–4. doi: 10.1111/j.1556-4029.2010.01572.x 20887355

[8] Storm BS Ludviksen JK Christiansen D Fure H Pettersen K Landsem A. Venous air embolism activates complement C3 without corresponding C5 activation and trigger Thromboinflammation in pigs. Front Immunol. (2022) 13:839632. doi: 10.3389/fimmu.2022.839632, PMID: 35371063 PMC8964959

[9] Branger AB Eckmann DM. Theoretical and experimental intravascular gas embolism absorption dynamics. J Appl Physiol. (1999) 87:1287–95. doi: 10.1152/jappl.1999.87.4.1287, PMID: 10517754

[10] Heckmann JG Lang CJG Kindler K Huk W Erbguth FJ Neundörfer B. Neurologic manifestations of cerebral air embolism as a complication of central venous catheterization. Crit Care Med. (2000) 28:1621–5. doi: 10.1097/00003246-200005000-00061, PMID: 10834723

[11] Brouns R De Surgeloose D Neetens I De Deyn PP. Fatal venous cerebral air embolism secondary to a disconnected central venous catheter. Cerebrovasc Dis. (2006) 21:212–4. doi: 10.1159/000090795, PMID: 16401887

[12] Shi L Zhang R Wang Z Zhou P. Delayed cerebral air embolism complicating percutaneous needle biopsy of the lung. Am J Med Sci. (2013) 345:501–3. doi: 10.1097/MAJ.0b013e31827bbe23, PMID: 23276901

[13] Faberowski LW. Incidence of venous air embolism during Craniectomy for Craniosynostosis repair. Anesthesiology. (2000) 92:20. doi: 10.1097/00000542-200001000-00009 10638894

[14] Soh M Hifumi T Isokawa S Iwasaki T Otani N Ishimatsu S. Persistent air embolism after blunt chest trauma with recovery to pre-existing consciousness level: a case report and literature review. Neurotrauma Rep. (2022) 3:38a–43a. doi: 10.1089/neur.2021.0052, PMID: 35112106 PMC8804252

[15] Kesieme E Feldmann M Welcker K Linder A Prisadov G. Cerebral infarct complicating traumatic Pneumatocele: a rare sequela following blunt chest trauma. Thorac Cardiovasc Surg. (2012) 60:e16–8. doi: 10.1055/s-0032-1304549 22549758

[16] Hwang SL Lieu AS Lin CL Liu GC Howng SL Kuo TH. Massive cerebral air embolism after cardiopulmonary resuscitation. J Clin Neurosci. (2005) 12:468–9. doi: 10.1016/j.jocn.2004.03.041, PMID: 15925785

[17] Abidine ZE Abdedaim H Omari D. Massive gas embolism secondary in the use of intraoperative hydrogen peroxide: still use to lavage with this liquid? Pan Afr Med J. (2013) 16:124. doi: 10.11604/pamj.2013.16.124.3499, PMID: 24839532 PMC4021982

[18] Dubey PK Singh AK. Venous oxygen embolism due to hydrogen peroxide irrigation during posterior Fossa surgery. J Neurosurg Anesthesiol. (2000) 12:54–6. doi: 10.1097/00008506-200001000-00011, PMID: 10636622

[19] Haller G Faltin-Traub E Faltin D Kern C. Oxygen embolism after hydrogen peroxide irrigation of a vulvar abscess. Br J Anaesth. (2002) 88:597–9. doi: 10.1093/bja/88.4.597, PMID: 12066743

[20] Park DH Chung YG Kang SH Park JY Park YK Lee HK. Arterial cerebral air embolism at the site of a spontaneous pontine hemorrhage in a patient receiving erroneous continuous positive pressure ventilation. Clin Neurol Neurosurg. (2007) 109:803–5. doi: 10.1016/j.clineuro.2007.06.006, PMID: 17681687

[21] Mishra R Reddy P Khaja M. Fatal cerebral air embolism: a case series and literature review. Case Rep Crit Care. (2016) 2016:1–4. doi: 10.1155/2016/3425321 PMC5011199 27635266

[22] Yu ASL Levy E. Paradoxical cerebral air embolism from a hemodialysis catheter. Am J Kidney Dis. (1997) 29:453–5. doi: 10.1016/S0272-6386(97)90209-2, PMID: 9041224

[23] Hysell MK. Cerebral air embolism after hemodialysis. J Emerg Med. (2015) 49:e27–8. doi: 10.1016/j.jemermed.2014.12.071, PMID: 25802160

[24] Segan L Permezel F Ch’ng W Millar I Brooks M Lee-Archer M. Cerebral arterial gas embolism from attempted mechanical thrombectomy: recovery following hyperbaric oxygen therapy. Pract Neurol. (2018) 18:134–6. doi: 10.1136/practneurol-2017-001828, PMID: 29288212

[25] Prasongsukarn K Borger MA. Reducing cerebral emboli during cardiopulmonary bypass. Semin Cardiothorac Vasc Anesth. (2005) 9:153–8. doi: 10.1177/108925320500900209 15920641

[26] Zink M Hainzl G Maier A Stadlbauer V. Cerebral air embolism after flushing a radial arterial line: a case report. J Emerg Crit Care Med. (2021) 5:28–8. doi: 10.21037/jeccm-20-174

[27] Santos JP Hamadeh Z Ansari N. Cerebrovascular accident secondary to paradoxical embolism following arteriovenous graft Thrombectomy. Case Rep Nephrol. (2012) 2012:1–3. doi: 10.1155/2012/183730, PMID: 24533201 PMC3914183

[28] Seeburger J Borger MA Merk DR Doll S Bittner HB Mohr FW. Massive cerebral air embolism after bronchoscopy and central line manipulation. Asian Cardiovasc Thorac Ann. (2009) 17:67–9. doi: 10.1177/0218492309102501, PMID: 19515884

[29] Oatis K Agarwal A Bruce-Tagoe C. Acute stroke from air embolism to the middle cerebral artery following upper gastrointestinal endoscopy. Radiol Case Rep. (2010) 5:359. doi: 10.2484/rcr.v5i1.359, PMID: 27307849 PMC4898213

[30] Hamilton-Farrell M Bhattacharyya A. Barotrauma. Injury. (2004) 35:359–70. doi: 10.1016/j.injury.2003.08.020 15037370

[31] Botez SA. Headache and cerebral venous air embolism. Neurology. (2007) 68:19–9. doi: 10.1212/01.wnl.0000236902.50380.ba, PMID: 17200486

[32] Bessereau J Genotelle N Chabbaut C Huon A Tabah A Aboab J. Long-term outcome of iatrogenic gas embolism. Intensive Care Med. (2010) 36:1180–7. doi: 10.1007/s00134-010-1821-9, PMID: 20221749

[33] Rubal BJ Leon A Meyers BL Bell CM. The “mill-wheel” murmur and computed tomography of intracardiac air emboli. J Am Assoc Lab Anim Sci. (2009) 48:300–2. PMID: 19476721 PMC2696835

[34] Muth CM Shank ES. Gas embolism. N Engl J Med. (2000) 342:476–82. doi: 10.1056/NEJM200002173420706 10675429

[35] Jorens PG Van Marck E Snoeckx A Parizel PM. Nonthrombotic pulmonary embolism. Eur Respir J. (2009) 34:452–74. doi: 10.1183/09031936.00141708 19648522

[36] McCarthy C Behravesh S Naidu S Oklu R. Air embolism: practical tips for prevention and treatment. J Clin Med. (2016) 5:93. doi: 10.3390/jcm5110093, PMID: 27809224 PMC5126790

[37] Shaikh N Ummunisa F. Acute management of vascular air embolism. J Emerg Trauma Shock. (2009) 2:180–5. doi: 10.4103/0974-2700.55330, PMID: 20009308 PMC2776366

[38] Leach RM Rees PJ Wilmshurst P. Hyperbaric oxygen therapy. BMJ. (1998) 317:1140–3. doi: 10.1136/bmj.317.7166.1140, PMID: 9784458 PMC1114115

[39] Moon RE. Hyperbaric treatment of air or gas embolism: current recommendations. Undersea Hyperb Med J. (2019) 46:673–83. 31683367

[40] Murphy BP Harford FJ Cramer FS. Cerebral air embolism resulting from invasive medical procedures. Treatment with hyperbaric oxygen. Ann Surg. (1985) 201:242–5. PMID: 3918516 10.1097/00000658-198502000-00019 PMC1250649

[41] Mathieu D Marroni A Kot J. Tenth European consensus conference on hyperbaric medicine: recommendations for accepted and non-accepted clinical indications and practice of hyperbaric oxygen treatment. Diving Hyperb Med J. (2017) 47:24–32. doi: 10.28920/dhm47.1.24-32 PMC6147240 28357821

[42] Mitchell SJ. Lidocaine in the treatment of decompression illness: a review of the literature. Undersea Hyperb Med J. (2001) 28:165–74. 12067153

